# Missing regulatory effects and viral triggers explored for childhood-onset asthma

**DOI:** 10.1016/j.xgen.2024.100652

**Published:** 2024-09-11

**Authors:** Hai Fang

**Affiliations:** 1 Shanghai Institute of Hematology, State Key Laboratory of Medical Genomics, National Research Center for Translational Medicine at Shanghai, Ruijin Hospital, Shanghai Jiao Tong University School of Medicine, Shanghai 200025, China

## Abstract

Missing regulatory effects of asthma genetic risks might be hidden within specific cell states. In this issue of *Cell Genomics*, Djeddi et al.^1^ uncover how airway epithelial cells, when activated by rhinovirus, influence genetic susceptibility to childhood-onset asthma, and this preview emphasizes the need to address these missing regulatory effects across diverse cell states.

## Teaser

Missing regulatory effects of asthma genetic risks might be hidden within specific cell states. In this issue of *Cell Genomics*, Djeddi et al. uncover how airway epithelial cells, when activated by rhinovirus, influence genetic susceptibility to childhood-onset asthma, and this preview emphasizes the need to address these missing regulatory effects across diverse cell states.

## Main text

**Figure 1:**
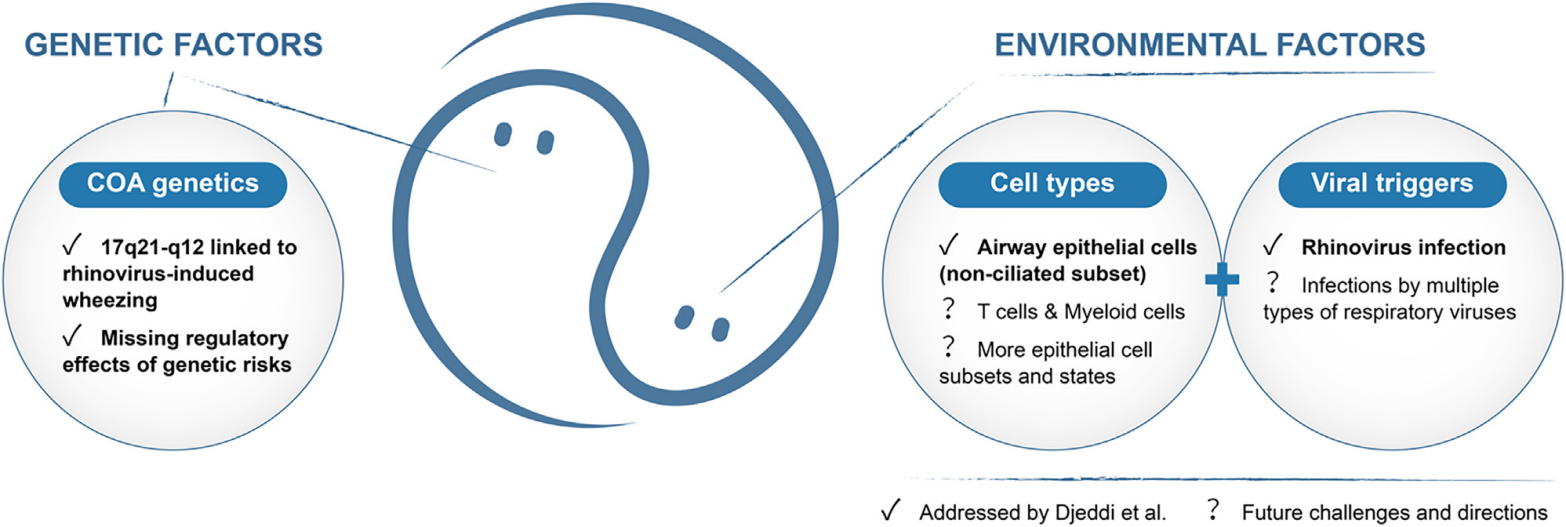
Interplay between genetic factors and environmental factors in COA The yin-yang-like illustration in the middle panel vividly depicts the interplay between child-onset asthma (COA) genetics and environmental factors (cell types + viral triggers; e.g., rhinovirus infection of non-ciliated airway epithelial cells) in driving COA, as explored by Djeddi and colleagues. In this preview, Fang outlines future challenges and directions for further resolving "missing regulatory effects" across diverse cell states; for example, identifying more subsets and states of COA-related cell types infected by multiple types of respiratory viruses that interact with COA genetic risk factors.

Asthma, one of the most common chronic non-communicable diseases, affects more than 300 million people worldwide, making it a major public health issue.^2^^,^^3^ The condition arises from complex interplay between genetic factors (what to inherit) and environmental factors (like allergens and pathogens), leading to different forms of asthma with various triggers. Advances in genomics and transcriptomics have provided deeper insights into the molecular and cellular mechanisms behind the disease. In this issue of *Cell Genomics*, Djeddi et al.^1^ explored the role of specific cells in our airways—airway epithelial cells—and how they interact with genetic susceptibility (inherited risk) and respiratory viruses, especially the rhinovirus, which often causes the common cold, in the development of childhood-onset asthma (COA; Figure 1). This preview will delve into the key messages of this study, focusing on: (1) disease genetic underpinnings and their missing regulatory effects; (2) the role of non-ciliated airway epithelial cells in linking rhinovirus to COA; and (3) future research directions to address upcoming challenges.

*Genetic underpinnings and their missing regulatory effects*. Genetics plays a significant role in asthma, accounting for 35%–95% of the overall risk. Genome-wide association studies (GWASs) have identified more than 150 genetic regions, or loci, associated with the disease.^4^^,^^5^ Many of these loci are in non-coding regulatory regions of the human genome, likely influencing gene regulation rather than coded proteins directly. One notable region on chromosome 17 (17q21-q12) has been closely linked to COA and rhinovirus-triggered wheezing.^6^ While it is difficult to pinpoint exactly how these non-coding regions contribute to COA risk, integrating GWAS data with context-specific functional genomics can help understand specific cell types, states, and molecular pathways involved. For instance, it has been discovered that chromatin regulatory elements specific to T cells, a type of immune cells, are more likely to undergo genetic changes that increase asthma risk,^7^ indicating that T cells are crucial in disease genetic susceptibility. However, there are still “missing regulatory effects”^8^—important genetic influences that remained undiscovered—that may lie within COA-related cell types other than T cells.

*Airway epithelial cells and respiratory viruses*. Airway epithelial cells, which form the first defense against inhaled substances like allergens and viruses, are vital in starting and modulating immune responses in the airways. Djeddi et al.^1^ investigated whether specific states of these airway cells could influence genetic susceptibility to COA and how they interact with rhinovirus, a virus known to trigger the disease. By analyzing gene expression data from epithelial cells under different activation conditions, including rhinovirus infection, they found that rhinovirus significantly increased the activity of genes related to COA. This effect was particularly evident in non-ciliated epithelial cells (i.e., less specialized cells in the airway lining), suggesting that these cells are key mediators in the genetic risk linked to rhinovirus-induced COA. This discovery supports the idea that rhinovirus might directly cause asthma in some children rather than simply being a biomarker for those who are already at risk. Although similar gene activity patterns were seen with influenza virus infection and cytokine activation, the response was not as specific as with rhinovirus. This points to a unique interaction between rhinovirus and asthma-associated genes, highlighting the specificity of this response. The study also implies that not all children who experience rhinovirus-induced wheezing will develop asthma; it appears that a combination of rhinovirus and a high genetic burden increases the likelihood of developing the disease. While T cells have long been recognized as important in asthma,^7^ this study emphasizes the unique role of airway epithelial cells in the disease. It suggests that asthma is a complex condition involving multiple cell types and molecular pathways. They also propose that myeloid cells in the respiratory tract could be another key cell type worth further study to resolve “missing regulatory effects” in COA.

*Implications for future therapeutic and preventive strategies, challenges, and directions*. Identifying specific genes and cell states that mediate the interaction between genetic risk factors and environmental triggers (like rhinovirus) opens new opportunities for developing targeted asthma therapies. For instance, therapies targeting molecular pathways activated in non-ciliated epithelial cells during rhinovirus infection might prevent asthma in children who are genetically at risk. The study also pointed out potential drug target genes within genetic regions associated with asthma. One example is *IL4R*, a gene specifically targeted by the drug dupilumab,^5^^,^^9^ which could lead to more effective therapies, potentially with fewer side effects compared to current therapies that broadly impact the immune system.^10^ Moreover, this study could lay the groundwork for new preventive strategies, such as developing a rhinovirus vaccine, to stop COA before it starts. Despite the progress made in this study, there are still challenges ahead. One major challenge is the reliance on *in vitro* infection models, which might not fully reflect what happens in a real living body. Another issue is that the study mainly used data from people of European ancestry, which could limit the generalizability to other populations. Future research should include more diverse populations and *in vivo* models to validate and expand their findings. Last but not least, there is still much work to be done in precisely elucidating how non-coding genetic regions influence gene regulation in airway epithelial cells under specific conditions. This task will require more detailed single-cell genomic profilings with larger samples and more comprehensive analyses of infections by different respiratory viruses. Doing so will enable additional subsets and states of epithelial cells to be identified as environmental factors that interact with genetic risk factors to drive COA.

In summary, Djeddi et al.^1^ provide valuable insights into the intricate interplay between human genetics, airway epithelial cells, and respiratory viruses in the development of COA (Figure 1). By highlighting the role of non-ciliated epithelial cells and key effector genes in increasing genetic risk, it paves the way for more targeted therapeutic strategies. However, further research is essential to fully understand molecular and cellular mechanisms and translate these understandings into effective clinical treatments. The future holds promise for developing more effective and personalized approaches to managing and treating asthma, ultimately improving the lives of millions of people affected worldwide. This research not only enhances our understanding of asthma but also emphasizes the importance of integrating genetic data with context-specific functional genomics. As we continue to explore “missing regulatory effects” of asthma genetic risks across diverse cell states, new therapeutic and preventive possibilities will emerge, offering hope for better management and prevention of this complex condition.

## Acknowledgments

The author is grateful to Fuyu Li for the insightful discussions and also wishes to acknowledge funding from the National Natural Science Foundation of China (32170663) and Innovative Research Team of High-Level Local Universities in Shanghai.

## Declaration of interests

The author declares no competing interests.
